# Influence of the Therapeutic Alliance on the Rehabilitation of Stroke: A Systematic Review of Qualitative Studies

**DOI:** 10.3390/jcm12134266

**Published:** 2023-06-26

**Authors:** Alejandra Heredia-Callejón, Patricia García-Pérez, Juan Antonio Armenta-Peinado, Miguel Ángel Infantes-Rosales, María Carmen Rodríguez-Martínez

**Affiliations:** 1 Department of Physiotherapy, Faculty of Health Sciences, University of Malaga, 29071 Malaga, Spain; alejandraherediacallejon2@gmail.com (A.H.-C.); mainfantes@uma.es (M.Á.I.-R.); 2 Occupational Therapy Department, Hospital Marítimo, Servicio Andaluz de Salud (SAS), 29620 Malaga, Spain; terapiaocupacional.patricia@gmail.com; 3 Biomedical Research Institute (IBIMA), 29590 Malaga, Spain

**Keywords:** therapeutic alliance, stroke, rehabilitation, recovery of function, qualitative research

## Abstract

The therapeutic alliance is a fundamental component of rehabilitation in order to achieve effective outcomes. However, what develops, maintains or hinders this relationship has not been sufficiently explored. The aim of this systematic review is to recognize the role of the therapeutic alliance in the neurological rehabilitation process. A search for articles was carried out in the databases PubMed, Web of Science, SCOPUS, CINAHL, APA PsycInfo, OTseeker and Dialnet. They were selected according to the eligibility criteria. Internal quality assessment of the articles was measured with the Consolidated Criteria for Reporting Qualitative Research (COREQ). The systematic review was registered in PROSPERO (ID 346523). The search in the databases identified 1596 articles, from which 9 different studies were finally included in the systematic review, reflecting the limitations of studies in this field. All studies are qualitative, from the point of view of the patients themselves, their relatives and healthcare professionals. The total sample of the included studies is 182 participants (96 health professionals, 76 users and 10 relatives). Semi-structured interviews, focus group discussions and written reflections were mainly used to collect the data. In conclusion, the therapeutic alliance can be an active component in the post-stroke rehabilitation process. Being recognized as a person, collaboration with the therapeutic team, empathy, empowerment, confidence, professional skills, maintenance of hope and the role of the family have been identified as aspects that can have an influence on the therapeutic alliance.

## 1. Introduction

Acquired brain injury is defined as any brain damage occurring after birth [1]. Causes can be traumatic, due to extrinsic injury, or non-traumatic, due to internal pathological processes, encompassing multiple diseases [1,2,3]. These include stroke, an important public health issue causing significant problems of long-term disability and considerably decreasing quality of life [4,5].

Findings indicate that rehabilitation plays an essential role in establishing functional performance and quality of life for people after stroke [6,7].

This process of neurological rehabilitation aims to achieve functionality, recovering or developing mental, physical and social capacities to their maximum potential [8]. To this end, the therapeutic alliance is considered an important aspect and a determinant of outcomes [9,10], as it has been related to functional development, productivity, self-awareness and emotional self-regulation [11], in addition to improving symptoms, increasing satisfaction, quality of life and psychological wellbeing [12]. Some people have even reported that the quality of the therapeutic relationship is more meaningful to them than the content of the therapy or its outcomes [13].

The therapeutic alliance or relationship aims to establish a consensus on goals with the patient and a personal bond between both parties [12]. It requires a person-centered approach [11], responding to the patient’s needs and improving the experience and outcomes of the individual’s recovery process [14,15].

Historically, the concept of alliance dates back to the Freudian period of psychodynamic theory [9,16]. Subsequently, this aspect has been recognized as essential in therapist–patient relationships by different psychologists from different theoretical perspectives [16,17,18]. 

According to Bordin, a renowned American psychology professor, the alliance is composed of three components: agreement on therapeutic goals, consensus on the tasks that are part of the therapy and the bond between patient and therapist [16]. His theoretical framework supports most of the studies that evaluate the therapeutic alliance and its effect on neurologicalrehabilitation [11].

In clinical praxis, health professionals adapt treatment through their knowledge in order to improve the quality of life and wellbeing of the individual [19,20], but it is the active participation of the individuals themselves that contributes to the relationship [19,21]. Results from physical rehabilitation in stroke show that there is a correlation between the therapeutic alliance and patient engagement, which is a facilitating factor for adherence [12]. Conversely, lack of engagement limits the increase in function and has an impact on quality of life [22].

Europe is becoming an actively aging continent to be considered, and as age increases, neurological disorders become more prevalent [4,23]. Moreover, the number of people suffering a stroke at a younger age is increasing [24], and there is evidence that health services will not be able to deal with this increase [4].

For this reason, this systematic review aims to conduct an exploration of the most recent literature to recognize the role of the therapeutic alliance in the neurological rehabilitation process. This concept plays an important role in healthcare, but research on this aspect in stroke rehabilitation is limited [11].

## 2. Materials and Methods

The development of this systematic review was guided by the “PRISMA 2020” statement, and its registration information is available [25].

### 2.1. Information Sources and Search Strategy

To carry out this systematic review, a search was performed in the databasesPubMed, Web of Science, SCOPUS, CINAHL, APA PsycInfo, OTseeker and Dialnet, using the descriptors “Therapeutic Alliance”, “Stroke”, “Outcome” and “Recovery of function”. The final search strategy focused on stroke due to a higher amount of published studies, which allowed finding more homogeneous results.

The search strategy used was the same in the different databases, obtaining different results through the possible applicable filters (see Table 1), limiting the search chronologically (2012–2022), by language and by type of study.

**Figure 1:**
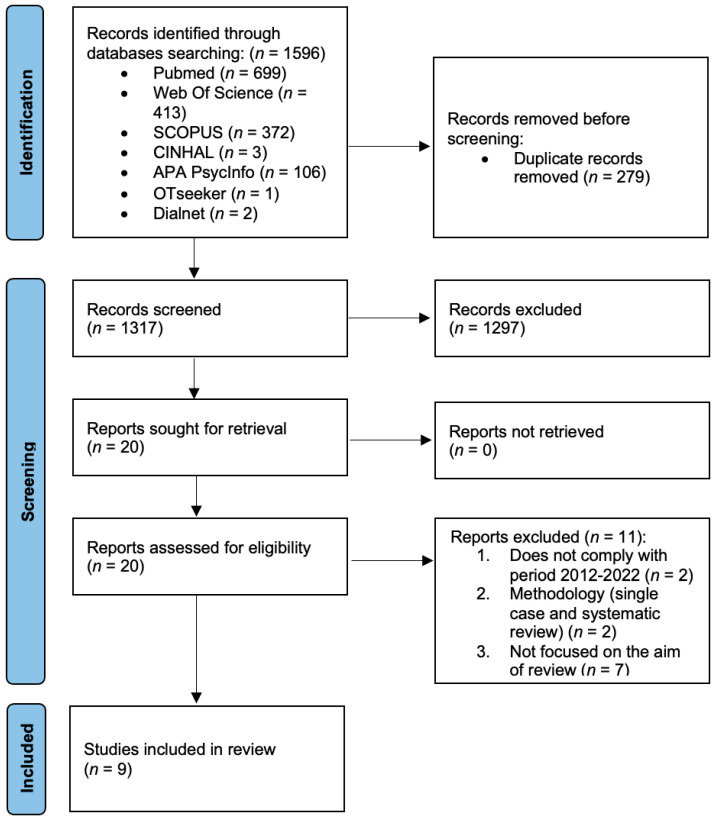
PRISMA flow diagram of study selection process.

To understand the basic procedures used in this systematic review and to analyze the screening and selection of the studies, a PRISMA flow diagram was completed (see Figure 1).

### 2.2. Quality Assessment of Included Studies

Internal quality assessment of the articles used in this systematic review was carried out using the Consolidated Criteria for Reporting Qualitative Research (COREQ). This instrument includes 32 items, covering the characteristics of the research team, the design and context of the study, the results of the study and the analysis and interpretation of the results [26].

### 2.3. Eligibility Criteria and Study Selection

Only studies published in English and in the last 10 years were selected for current data, including those published from January 2012 to January 2022. In terms of design, only qualitative studies that included exploratory, ethnographic, grounded theory, constructivist, interpretative action or interpretive description methodology were accepted. The only studies included were those that focused on acquired brain injury and the therapeutic alliance, in a physical, cognitive, language or functional rehabilitation context, with the user or their relatives.

On the other hand, studies that focused on other neurologic pathologies and one case study were excluded, as well as studies that focused on the therapeutic alliance within pharmacological therapies, because direct interaction with the therapist may be less frequent compared to physical, cognitive, language or functional rehabilitation carried out by health professionals.

## 3. Results

The final screen of the systematic review contains nine articles, which are presented in Table 2. In addition, the authors, year, type of study, study objective, sample, instruments used, some intervention details, main results and internal quality of the results are also briefly presented, considering the qualitative research tool mentioned above. The total sample of the included studies is 182 participants (96 health professionals, 76 users and 10 relatives).

The quality of the studies included in the review has an average score of 25/32, which indicates an optimum value.

Most of the criteria that have not been fulfilled are related to the personal characteristics of the research team, such as their credentials, their occupation at the time of the study and their experience and training.

Other lacking criteria are related to the study design, in cases where we did not find information about presence of non-participants at the time of the interview and items related to data collection, such as whether interviews were repeated and whether data saturation was reached.

## 4. Discussion

The results of this systematic review show the characteristics associated with the therapeutic alliance in the post-stroke rehabilitation process from the point of view of healthcare professionals, patients themselves and their relatives.

Qualitative research studies explore perspectives and experiences of individuals [27], deepening the understanding of the post-stroke adaptation process of survivors and guaranteeing a person-centered practice [28].

Different aspects shared in the studies analyzed have been identified, such as the recognition of personhood, which is considered a process that builds the foundations of trust and the therapeutic alliance [11,29,30]. Differences are found between studies that support the breaking of the therapeutic gap [31] and others that value the preservation of professional limits [11,29,32].

According to some studies, patients feeling listened to and understood will increase personal connection and the perception of support [11,30,31,32,33], while for others, this relationship may create dependence [29].

Finally, we also highlight factors such as hope [29,33,34] and the role of the family as contextual shapers of the development of the therapeutic alliance [11,29], as compared to studies in which the single relationship between professional and patient [11] is prioritized.

Based on the different studies consulted, we divide the present discussion into different sections on topics considered basic aspects in the therapeutic alliance that need to be addressed. The topics presented below are “Being recognised as a person”, “Empathy, emotional bonding and trust”, “Collaboration” (with the subtopics “Individualising rehabilitation”, “Control and empowerment” and “Role of health professionals”), “Maintaining hope” and “The family”.

### 4.1. Being Recognised as a Person

Recognition of patients’ personhood is apparently one of the first processes that promotes trust and builds the basis for the development of the therapeutic alliance [11,29,30], which originates from a connection on a human level [30]. Several people valued identity preservation by healthcare staff [32]. This quality has not always been prioritized, as some professionals consider it incongruent with patients’ expectations of their recovery process [29].

Self-revelation by professionals appears to be a feature that helps build recognition and bonding in the dyad [11,29,31,32], often being perceived as a way of breaking down barriers between both parties [11]. The therapeutic gap allows people to feel more comfortable and connected to the community [31]. However, others maintain their stance on the preservation of professional barriers [29]. In addition, there are patients who recognize the need for these limits [11], and some with fewer rehabilitation needs express satisfaction with this division approach, simply requiring therapists to focus on treatment [32], in order to “run away home” [11].

Hersh et al. [35] also highlight informal assessment as a more person-centered method of working in the early post-stroke period, the first point of contact with patients, during which professionals want to establish and facilitate interpersonal relationships.

Many professionals described the difficulty of this approach due to the hospital environment and policies, which do not allow for an in-depth relationship due to time and resource constraints [29]. However, professionals engaging in getting to know the person seems a crucial process in rehabilitation [33].

### 4.2. Empathy, Emotional Bonding and Trust

Close therapeutic alliances were described as open and relaxed, without any judgement from the staff [32]. In the study of Walder and Molineux [34], some people report that they perceived a lack of understanding of experience, thoughts and emotions on the part of healthcare staff.

The feeling of being listened to and understood is seen as critical to mitigating isolation and building trust [32], as well as increasing personal connection [11] and the perception of support [31]; particularly in the early stages of rehabilitation, during which people feel more vulnerable and report needing more than professionalism [30,32].

Although forming bonds is seen as a vital aspect of engagement, it has been felt that if bonds become too close, it can create dependency [29]. Therapists reported that deep connection and trust are dependent on time and frequency of contact [29]. In the study of Williams and Douglas [31], patients’ sense of comfort was related to the friendliness of professionals, and they highlighted the importance of a welcoming and positive first contact.

To empathize with patients, professionals highlighted the value of showing an understanding of the impact of stroke on a person’s life, through listening and normalizing the experience [29].

Even goal setting requires some work on an emotional level by therapists that often seems extraneous to professional value but is a crucial part of person-centered care [36].

A trusting context allows for an open and transparent space that facilitates getting to know each other and implies that patients feel safe in the interaction. In addition, trust promotes engagement in the recovery process [33].

This can be affected by the sense of unfamiliarity generated by healthcare staff, as many patients do not come to understand their diagnosis and the recovery process and are often not offered explicit information [32], creating a sense of uncertainty in the early stages [33].

### 4.3. Collaboration

Collaboration appears to be based on building a mutual consensus on the individual’s environment and characteristics [11]. Many report that it is dependent on the therapist’s ability to get to know the person and identify their needs [32] or meaningful goals [34] in order to acquire a shared approach [11].

Therapeutic alliance is clearly related to appropriate connection and collaboration in the recovery process [31,32].

Collaborating in the relationship seems to imply reciprocity [11] and investment [33]. Reciprocity shows the relationship between engagement and trust [33], and personhood recognition sets the foundation for its development [29]. On the other hand, investment refers to the dedication and involvement at the emotional level experienced by dyad members in the recovery process [33].

In the study of Kayes et al. [33], the professionals reported difficulty in engaging if patients are not engaged, and the individuals acknowledged their active role in the collaboration.

Furthermore, when exploring individuals’ experiences, they expressed the importance of being able to be part of the team, feeling able to contribute to it [31].

Time limitations and the pressure to achieve goals within hospital policies also influenced engagement, with it being more favored in a home environment characterized by a relaxed atmosphere [29].

### 4.4. Individualising Rehabilitation

Many highlight as an essential factor in the therapeutic alliance the individualization of rehabilitation according to the needs of the patient [11,29,31,32,34,36].

In the study of Lawton et al. [29], they express it as being responsive and adapting behaviors to meet the person’s needs. In this process, the knowledge of the patient on the part of the healthcare staff comes into play [29,33]. Mutual understanding enables the skills of professionals to be adapted to the needs and preferences of patients [11].

On one hand, there are information needs, as expressed in the studies of Lawton et al. [29], Lawton et al. [32] and Walder and Molineux [34], as there are patients who need to understand the goal they are working on in the rehabilitation exercises. Information needs even include information about the diagnosis, the prognosis and their process, as expressed by Lawton et al. [32].Transition points such as discharge or assessment for admission to rehabilitation were key points where participants noted a lack of information [34], and adapting this to their understanding was valued by patients [31].

On the other hand, there was also the need for adaptation, highlighted in the study of Kayes et al. [33].Practitioners need to know where the pressure limit is, balancing a challenge without creating a sense of frustration due to functionality problems.

Underpinning this is the ability of therapists to respond and adjust tasks accordingly [32].

A non-adaptive outcome could lead to disengagement of the patient in their process [11,34] and affect their engagement [32].

### 4.5. Control and Empowerment

These aspects are seen as an integral part of the development of the therapeutic relationship, as professionals take on an accompanying role in the rehabilitation process [29] and patients want to be empowered [36], a dimension included in the dynamic conceptualization of the alliance [37].

Taking ownership of the recovery process is relevant for patients [31,32,34]. Participants in the study of Walder and Molineux [34] reported not feeling in control of important decisions and feeling excluded from preferences about their care, with no consideration of their emotions or needs.

On the other hand, some prioritized therapists taking on this responsibility [11,32] and others reported being unprepared due to cognitive, emotional or psychosocial factors [36], particularly in the early stages of stroke [29].

The fact that professionals place trust in patients’ ability to be active agents in the process, taking risks and knowing their abilities, seems to be an important aspect for them [33]. In addition, professionals described feeling more relaxed with people who were in control and actively involved in the relationship [29].

The findings of dissatisfied needs suggest that the level of collaboration and empowerment of the individual may not always be optimal [38]. Alliances distinguished by imposition accentuate the professional–patient division and have the potential to damage self-esteem [32].

### 4.6. Role of Health Professionals

Professionals’ skills also interact to dictate the success of the alliance [11,29]. Patients described that feeling heard and understood and understanding information derived from their recovery process depended on the therapist’s communication skills [31,32]. In addition, other studies add knowledge as a basis for adapting rehabilitation according to the characteristics of the individual [29,32,33].

Personal engagement and advanced communication skills can add significant value to therapeutic connections, but many professionals are not prepared for this [36]. Some therapists report feeling poorly equipped, lacking the necessary skills to promote behavior change and intervene in patients’ engagement and motivation [29].

On the other hand, it is important for individuals to know the rehabilitation team, their background, their experience and their qualifications, as it seemed to establish trust within the dyad [33].

### 4.7. Maintaining Hope

There is a discomfort for professionals with setting ‘unrealistic’ goals for patients, creating an acknowledged emotional tension due to conflict between the biomedical model and the person-centered perspective [36]. In the study of Lawton et al. [29], several speech therapists reported that there is a possibility of disengagement and the creation of conflict with the patient if conflicting expectations are not managed. At the same time, such a realistic approach can damage hope, and so‘walking the line’ is recommended, which involves balancing what are perceived as realistic expectations with hope. 

False hope is correlated with a lack of skills or explicit knowledge on the part of health professionals, who do not believe in influencing this concept [29]. However, health professionals can intervene in it in a meaningful way, and it is considered an important aspect for patients in their recovery process, as they identify it as an emotional motivator related to quality of life and influencing their engagement [33,34].

Findings from previous studies already identify the importance of aligning expectations and maintaining hope during alliance building [39,40,41,42]. In the study of Walder and Molineux [34], patients described that, to protect this maintenance of hope, it is necessary to know when not to press to safeguard the person’s psychological state and preserve optimism in the dyad.

### 4.8. The Family

Recently, the dynamic concept of the alliance has also been extended to include the family dimension [37], in which rehabilitation recognizes individuals and their relatives as team members involved in decisionmaking [43].

As some studies show, the therapeutic alliance is not limited to the professional–patient dyad, and the family connection becomes vitally important [11,29]. It was recognized as a context shaperthat facilitates or impedes the development of the alliance [11,29]. However, others prioritize the exclusive therapeutic relationship between healthcare professionals and patients [11].

Several studies have shown that a good alliance with the family influences outcomes, making it another crucial component in rehabilitation [43].

We have been able to explore the characteristics of the therapeutic alliance during the post-stroke recovery process, but more research is needed on the role of the therapeutic alliance in rehabilitation. For example, it would be valuable to increase the sample size of studies in order to build on the current knowledge base. It would also be useful to explore the relationship of the therapeutic alliance with adherence, engagement and rehabilitation outcomes. 

This study has potential limitations. When carrying out searches in the different databases, a seemingly large number of results were found, which has then been significantly reduced by the exclusion criteria of this review. This is justified, as there is a considerable amount of research on the therapeutic alliance, but most of it is associated with pharmacological therapies, without focusing on the process of functional rehabilitation after stroke. Furthermore, following the difficulties experienced in the early stages of the research process, studies with less scientific evidence have been used, justified by the absence ofquantitative results.

## 5. Conclusions

The therapeutic alliance concept has been explored in recent qualitative studies, in which patients, their relatives and health professionals have recognized that it can be an active component in the rehabilitation process after stroke.

The research involved shows us the characteristics that can influence the therapeutic alliance, such as being recognized as a person; collaboration with the health professional, including qualities such as empathy, trust, empowerment and professional skills; maintenance of hope; and the role of the family.

Despite all the findings, the progress in the literature and the knowledge we have that the therapeutic alliance is of significant value in healthcare, research on this aspect of stroke rehabilitation is limited [11]. Further understanding of the role this concept plays in the process of recovery and adaptation after stroke may be helpful in working with individuals to optimize rehabilitation outcomes. Therefore, we suggest continuing with research and carrying out studies on a larger scale, in order torecognize the therapeutic alliance as a determinant in the recovery process and to be able to achieve better outcomes and increase quality of life.

Furthermore, the relationship of the therapeutic alliance to adherence and the development of assessment tools to measure this concept could be of interest. The first accessible and theoretically robust measure of therapeutic alliance has recently been developed, called “Aphasia and stroke therapeutic alliance measure (A-STAM)”. The sample size and the exclusion of people with more severe language abilities and family members are some of its limitations [44]. Therefore, modifying them may become of significant importance for research in order to further study and understand therapeutic alliance.

## Figures and Tables

**Table 1:** Databases and search results.

Search Strategy: (Therapeutic Alliance) or (Therapeutic Relationship) and (Stroke) and (Outcome) or (Recovery)		
Databases Consulted	Filters	Results
PubMed	Results by year: 2012–2022 Language: English Document type: Randomized Controlled Trial	699
Web of science	Results by year: 2012–2022 Language: English Document type: Article	413
SCOPUS	Results by year: 2012–2022 Language: English Document type: Article	372
CINAHL	Results by year: 2012–2022 Language: English Document type: Randomized Controlled Trial	3
APA PsycInfo	Resultsbyyear: 2012–2022 Language: English Document type: Clinical trial	106
OTseeker	Application of filters is not allowed	1
Dialnet	Results by year: 2012–2022 Language: English Document type: Article	2

**Table 2:** Overview of included studies.

First Author and Year	Type of Study	Main Research Aim	Participants (*N*)	Instruments	Characteristics of the Intervention	Outcomes	Quality of the Studies
Mudge et al. (2014) [27]	Qualitative ethnographic study	Understand the shared conflictual response to and discomfort with person-centered rehabilitation in the rehabilitation context.	*N* = 2 physiotherapists	10 written reflections and 5 joint discussions	Reflections following a coaching session. Discussion after reading them. Agreeing on the next topic and carrying out literature research and reading.	Biomedical dominance places the health professional as the expert, limiting the value they place on gaining the patient’s perspective and a collaborative working engagement.	COREQ: 26/32
Lawton et al. (2018a) [28]	Qualitative exploratory study	To explore perceptions and experiences of developing and maintaining therapeutic alliances in aphasia rehabilitation post-stroke.	*N* = 22 speech and language therapists (24–58 years)	Semi- structured interviews	Included relationship, collaboration, expectations and roles, motivation, experience and family involvement. · Recordings and transcriptions · Inductive analysis	Therapists use multiple strategies to develop alliances with people with aphasia in order to establish the basis for therapeutic work. In addition, there are external variables that interact to impede or facilitate the development of the alliance.	COREQ: 25/32
Hersh et al. (2018) [29]	Qualitative study	Explore interactions during informal assessment, the balance of interactions and their impact on the development of the therapeutic relationship.	*N* = 3 men with aphasia (74, 48 and 80 years)	Assessment sessions	· Recordings and transcriptions · Reflections on the sessions	Informal assessment by clinicians can go beyond the choice of non-standardized tasks and materials. It also involves a number of interactive features aimed at normalising a set of potentially uncomfortable and challenging activities, forming a balance in interactions and facilitating relationships.	COREQ: 22/32
Lawton et al. (2018b) [30]	Qualitative study	To investigate the experiences and reflections of people with aphasia about building and maintaining therapeutic alliances in rehabilitation.	*N* = 18 people with aphasia (45–88 years)	Semi- structured interviews	· Recordings and transcriptions · Field notes · Inductive thematic analysis	The therapist’s ability to adapt the alliance to the individual’s preferences and needs dictated the perceived success of the alliance. Positive alliances have the potential to stimulate both hope and engagement.	COREQ: 26/32
Walder et al. (2020) [31]	Constructivist grounded theory qualitative study	To explore how stroke survivors perceive their relationship with the healthcare team as they adjust to life after stroke.	*N* = 6 (34–76 years)	Semi- structured interviews	Open-ended and probing questions · Transcriptions · Simultaneous data collection and analysis	Positive aspects of relationships were recognized, such as the provision of information, professional practice, hope and emotional support.	COREQ: 25/32
Bishop et al. (2021) [11]	Qualitative study with interpretative description	To explore the basic components of a therapeutic alliance and the factors that are perceived to impact its development in a stroke rehabilitation unit.	*N* = 17 (29–76 years) Users (*N* = 10) Professionals (*N* = 7)	Semi-structured interviews and focus group discussions	Individual and focus group interviews · Recordings and transcriptions · Field notes · Conventional content analysis	Personal connection, professional collaboration and collaboration between family and clients appear to be the most prominent core components of the relationships. Given the value placed on it by participants, determining and addressing each client’s therapeutic relationship can augment rehabilitation processes and outcomes.	COREQ: 24/32
Kayes et al. (2021) [32]	Qualitative exploratory study	Identify key processes for engagement in stroke rehabilitation.	*N* = 19 users (46–83 years)	Semi- structured interviews	Individual interviews · Recordings and transcriptions · Collaborative and iterative analysis. ·Coding, note taking, diagramming and group discussions.	Engagement is a complex, nuanced, responsive, flexible and bidirectional process. The development of connections appears to be central to engagement, and connections take many forms. The most fundamental was the therapeutic connection between the person with stroke and their professional.	COREQ: 27/32
Williams et al. (2021) [33]	Qualitative study with grounded theory methodology	To obtain an understanding of the therapeutic alliance in community rehabilitation from the perspective of adults with traumatic brain injury and their relatives.	*N* = 6 (30–45 years) Users (*N*= 3) Relatives (*N* = 3)	Semi- structured interviews	Individual interviews · Recordings and transcriptions · Field notes	Therapeutic alliance is an important concept for the rehabilitation experience of people with traumatic brain injury and their relatives. It involves being recognized as an individual, working together and feeling personally connected.	COREQ: 24/32
Gordon et al. (2022) [34]	Appreciative action research study	Describe the processes involved in the creation of meaningful relationships in stroke units.	*N* = 89 (46–95 years) Professionals (*N* = 65) Users (*N* = 17) Relatives (*N* = 7).	Informal observation, informal discussions, focus groups and semi- structured interviews	Four stages: discover, envision, design and integrate. Iterative cycles of feedback, reflection and evaluation.	The processes that support human connections in practice were sensitising to humanize relational knowledge; valuing, reflecting on and sharing relational experiences; and having the freedom to act, enabling human connections.	COREQ: 23/32

## Data Availability

For additional information, please contact with María Carmen Rodríguez-Martínez (marrodmar@uma.es).
